# Successful Intravascular Correction of Intratumoral Pseudoaneurysm by Erosion of the Aorta in a Patient with Thoracic Giant Cell Tumor of Bone Responding to Denosumab

**DOI:** 10.1155/2015/626741

**Published:** 2015-10-27

**Authors:** Natalia M. P. Fraile, Diego Toloi, Ceci O. Kurimori, Adriana R. B. Matutino, Alberto Codima, Veridiana P. Camargo, Olavo Feher, Rodrigo R. Munhoz

**Affiliations:** ^1^Instituto do Câncer do Estado de São Paulo, Faculdade de Medicina da Universidade de São Paulo, São Paulo, SP, Brazil; ^2^Hospital das Clínicas, Faculdade de Medicina da Universidade de São Paulo, São Paulo, SP, Brazil

## Abstract

Giant cell tumor of bone (GCT) is a rare, locally aggressive neoplasm characterized by the presence of giant cells with osteoclast activity. Its biology involves the overexpression of the *Receptor Activator of Nuclear Factor kB Ligand* (RANKL) by osteoclast-like giant cells and tumor stromal cells, which has been shown to be an actionable target in this disease. In cases amenable to surgical resection, very few therapeutic options were available until the recent demonstration of significant activity of the anti-RANK-ligand monoclonal antibody denosumab. Here we present a case of a patient with advanced GCT arising in the spine, recurring after multiple resections and embolization. Following initiation of denosumab, which resulted in unequivocal clinical improvement, computed tomography of the chest done for reassessment purposes revealed an intratumoral pseudoaneurysm by erosion of the aorta, further corrected by endovascular approach and stent placement. Patient had an unremarkable recovery from the procedure and continued benefit from therapy with denosumab and remains on treatment 24 months after the first dose.

## 1. Introduction

Giant cell tumor of bone (GCT) is a rare osteolytic neoplasm characterized by the presence of giant cells with osteoclast activity [1, 2]. Although distant metastases seldom occur, significant morbidity and functional disability can result from local aggressiveness, and multiple local relapses can occur during the course of this disease [2, 3].

The pathogenesis of GCT involves the overexpression of the* Receptor Activator of Nuclear Factor kB Ligand* (RANKL) by osteoclast-like giant cells, a characteristic shared by mononuclear cells in the tumoral stroma [2, 4–6]. Osteoblasts secrete RANKL, involved in osteoclast precursors activation and subsequent osteolysis, which promotes release of bone-derived growth factors, such as insulin-like growth factor-1 (IGF1) and transforming growth factor-beta (TGF-beta), and increases serum calcium levels. RANKL is a central mediator of osteoclast activity and recruitment of precursors that differentiate into multinucleated osteoclast-like giant cells and is directly involved in the pathogenesis of GCT [2, 4–6].

Denosumab, a monoclonal antibody, binds to RANKL, blocks the interaction between RANKL and RANK (a receptor located on osteoclast surfaces), and prevents osteoclast formation, leading to decreased bone resorption and increased bone mass in osteoporosis. In solid tumors with bony metastases, RANKL inhibition decreases osteoclastic activity leading to decreased skeletal related events and tumor-induced bone destruction. In giant cell tumors of the bone (which express RANK and RANKL), denosumab inhibits tumor growth by preventing RANKL from activating its receptor (RANK) on the osteoclast surface, osteoclast precursors, and osteoclast-like giant cells. Denosumab has demonstrated substantial efficacy in this disease and is currently approved by several regulatory agencies for the treatment of patients with advanced GCT [2, 4–7].

We present a case of a patient with a recurrent spinal GCT treated with denosumab, with significant clinical and radiologic response. Clinical course during treatment with denosumab was complicated by an intratumoral pseudoaneurysm resulting from erosion of the aorta, successfully corrected by endovascular approach.

## 2. Case Presentation

**Figure 1:**
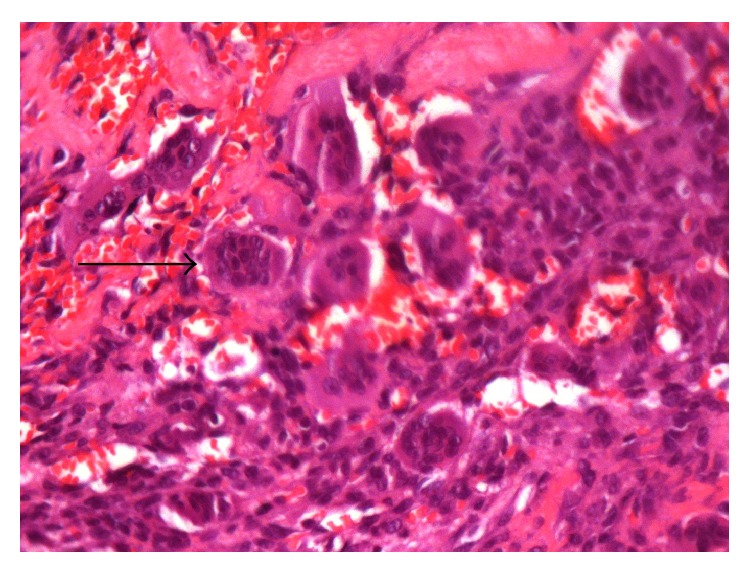
Hematoxylin and eosin-stained tumor tissue depicting osteoclast-like giant cells.

**Figure 2:**
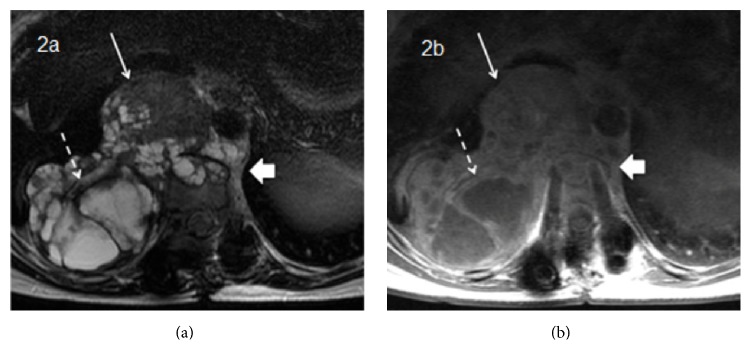
Baseline (pretreatment) MRI findings on T2-weighted (a) and postgadolinium T1-weighted (b) images showing a mass arising from the vertebral body (wide arrow), with a heterogeneous soft tissue component with solid (narrow arrow) and cystic areas (dashed arrow).

**Figure 3:**
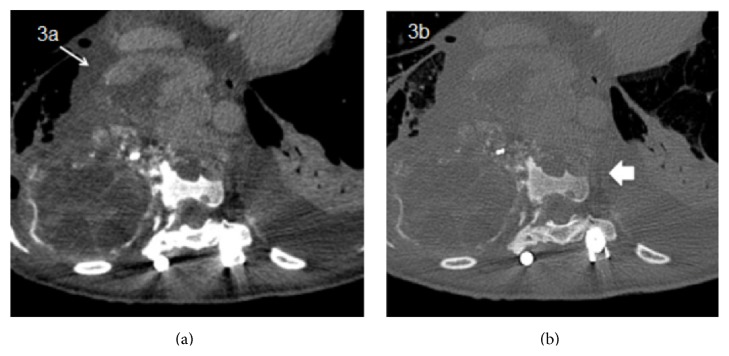
Postcontrast CT on soft reconstruction filter (a) and hard reconstruction filter (b), showing a lytic bone lesion arising from the vertebral body (wide arrow) with a large soft tissue component (narrow arrow).

**Figure 4:**
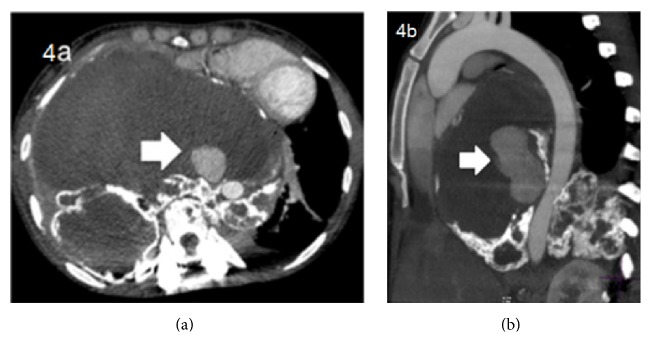
Postcontrast/arterial phase (a) and oblique reformatting (b) showing a pseudoaneurysm arising from the thoracic aorta and more prominent areas of calcification consistent with response to treatment.

**Figure 5:**
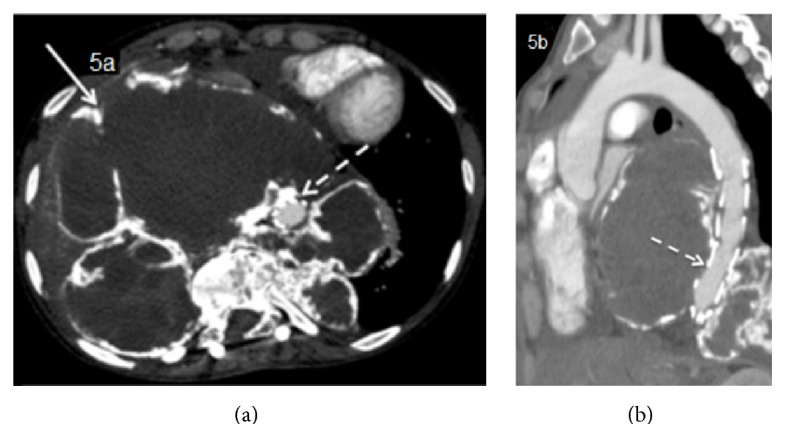
Postcontrast/arterial phase (a) and oblique reformatting (b) after successful placement of aortic endovascular stent graft and repair of the pseudoaneurysm.

**Figure 6:**
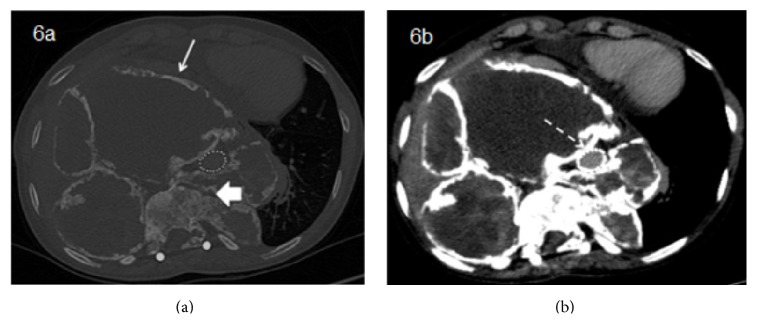
Long-term follow-up CT after continued treatment with denosumab showing more prominent calcification (wide arrow) and reduction of the soft tissue component (narrow arrow). The aortic stent is seen, without extravasation of contrast (dashed arrow).

A 29-year-old female initially presented with progressive back pain and parenthesis/paresis radiating to the left leg. Initial workup revealed an expansive process arising in the 10th thoracic vertebral body with invasion of soft tissues/epidural space. She initially underwent spine decompression/fixation and partial resection of the mass. Pathology was consistent with GCT of bone, with exuberant osteoclast-like giant cells, as shown (Figure 1). Despite initial control, she developed multiple local recurrence and underwent repeated resections/embolization during subsequent years. Ultimately, she was referred to medical oncology for consideration of additional systemic therapy after progression on zoledronate. At baseline, patient had significant thoracic pain and was dependent on oxygen due to a large mass partially obstructing the right bronchus (Figures 2 and 3). Denosumab 120 mg given every 28 days was started, with loading doses on days 8 and 15 of the first cycle. After 3 cycles, patient had remarkable clinical improvement, no longer requiring analgesics or oxygen therapy. Restaging scans revealed sclerosis/ossification and reduction of the soft tissue component, consistent with response to treatment. Nevertheless, CT after contrast/arterial phase disclosed a pseudoaneurysm arising from the thoracic aorta with focal extravasation of contrast (Figure 4). The patient was admitted to the hospital and underwent endovascular placement of a stent (Figure 5). Treatment with denosumab was resumed, with continued symptomatic and radiologic improvement on subsequent evaluations (Figure 6) and no significant toxicities, with ongoing treatment and sustained response 24 months after first dose of denosumab.

## 3. Discussion 

GCT is a rare, potentially aggressive primary bone tumor that usually affects young adults, with a slight female predominance, arising most frequently in epiphyses of long bones and sporadically in the axial skeleton [1–3]. Although distant metastases are rare, occurring in less than 3–6% of the patients and typically affecting the lungs, local recurrences are often seen following locoregional approaches [3, 8].

Radiographically, GCT is characterized by a lytic appearance with a nonsclerotic border, occasionally showing cortical expansion and pathological fracture [2, 3]. On MRI, GCT typically shows low to intermediate signal intensity on T1- and intermediate to high signal intensity on T2-weighted sequences, with early enhancement followed by contrast washout after administration of gadolinium [2, 3].

Although complete excision remains the mainstay of treatment, surgical resection can be complicated by significant morbidity and locoregional recurrence. For nonsurgical candidates, alternatives include bisphosphonates, embolization, radiation therapy, and, more recently, denosumab [2, 4]. RANK/RANKL-dependent signaling has been demonstrated in different components of the tumoral mass, including mesenchymal stromal cells, mononuclear osteoclast precursors, and osteoclast-like giant cells, and targeting RANKL using denosumab has been shown to be a successful strategy [4–7]. Across different clinical trials, denosumab, a fully human monoclonal antibody, resulted in clinical, radiologic, and histological responses, leading to disappearance of giant cells on posttreatment biopsies and sustained disease control [4–7]. In a single-arm, phase II study, 37 patients were treated with denosumab 120 mg monthly, with loading doses on days 8 and 15 of the first month [4]. Among 35 evaluable patients, 86% met tumor response criteria, defined as elimination of at least 90% of giant cells by histopathology or no radiological progression up to week 25, including all patients (*n* = 20) assessed by posttreatment biopsy. Stromal expression of RANKL was also shown to decrease after elimination of the giant cells. Responses were accompanied by remarkable clinical improvement and pain control in the majority of patients [4], and clinically relevant decrease in pain was also reported in a separate prospective study investigating clinical outcomes of patients treated with denosumab [6]. In a subsequent phase II trial including 282 patients with GCT, objective responses occurred in 72% of 190 patients included in imaging analysis (25% by modified RECIST, 96% by EORTC criteria, and 76% by inverse Choi criteria), with a median time to objective response of 3.1 months [5]. Only 1% of the patients were primarily refractory to denosumab and had disease progression upfront [5]. Of note, activity of denosumab has also been demonstrated in patients with metastatic GCT [9]. Potential adverse events, although rare, include osteonecrosis of the jaw (1-2%), hypocalcaemia, hypophosphatemia, and pain in the extremities [4–7].

The case herein reported illustrates a situation in which excellent response to treatment resulted in locoregional complications due to the size and anatomical location of a large, thoracic GCT. Response to denosumab is typically characterized by sclerosis and reconstitution of cortical bone [2], findings that were observed in this case during the course of treatment. In a recently published phase 2 study published by Ueda et al. including 17 patients with GCT treated with denosumab [6], two cases of treatment-related grade 3 pneumothorax were reported. Therefore, continuous surveillance for potential complications even in the setting of response is advised during the treatment of patients with GCT, since local aggressiveness, large dimensions, and extension into surrounding tissues frequently characterize the clinical course of these tumors. Endovascular techniques using a stent graft represent less invasive alternatives to open surgical repair in individuals with aortic aneurysms [10, 11]. In a prospective study, endovascular repair was associated fewer late reintervention rates and similar long-term outcomes when compared with open surgical approaches in patients with vascular complications affecting the descending thoracic aorta [11].

In conclusion, GCT remains a challenging condition and a multidisciplinary approach is essential for the successful management of this rare disease and denosumab emerged as an active treatment for patients with advanced disease. The optimal treatment duration and schedule (continuous versus intermittent) of denosumab and its role in the neo- and adjuvant setting still need to be clarified.
